# Footwear characteristics and foot problems in community dwelling people with stroke: a cross-sectional observational study

**DOI:** 10.1080/09638288.2022.2102679

**Published:** 2022-08-14

**Authors:** Dorit Kunkel, Louis Mamode, Malcolm Burnett, Ruth Pickering, Dan Bader, Margaret Donovan-Hall, Mark Cole, Ann Ashburn, Catherine Bowen

**Affiliations:** a School of Health Sciences, University of Southampton, Southampton, UK; b Faculty of Medicine, University of Southampton, Southampton, UK

**Keywords:** Indoor and outdoor shoes, poorly fitting shoes, foot problems, stroke, footwear

## Abstract

**Purpose:**

To explore footwear characteristics and foot problems in community dwelling people with stroke as most research to date focused on the general elderly population.

**Methods:**

Thirty people with mild to moderate stroke (nine men, mean age 68, mean time since onset 67 months) attended a single session to assess footwear and foot problems using established podiatry foot (wear) and ankle assessments.

**Results:**

Most participants wore slippers indoors (*n* = 17, 57%) and walking shoes outdoors (*n* = 11, 37%). Over half wore unsupportive ill-fitting shoes indoors and 47% of outdoor shoes fitted badly. All participants had foot problems (mean 6.5 (3.1), 95% CI: 5.4–7.7), including impaired single limb heel raise (93%), reduced range of movement (77%), sensation (47%), and muscle strength (43%). Many had foot-pain, hallux valgus (both 50%), or swollen feet (40%). Foot problems were associated with reduced balance confidence, activity, and community participation (all *p* < 0.05). A greater proportion of fallers (13/16) than non-fallers (4/14) reported foot problems (*p* = 0.029).

**Conclusions:**

Many community-dwelling people with stroke wore poorly fitting shoes; all had foot problems. Foot problems were linked to reduced mobility. Finding more effective pathways to support people with stroke to select supportive, well-fitting indoor and outdoor footwear is indicated.

**Implications for rehabilitation**
- People with stroke often wear unsupportive ill-fitting shoes and experience foot problems.
- Assessment of foot problems and footwear advice should be considered during stroke rehabilitation particularly when interventions target fall prevention or improvements in balance and mobility.
- Information on appropriate footwear and signposting that new shoe purchases should include measuring feet to ensure a good fit is recommended.

## Background

Inappropriate footwear such as walking barefoot, in socks, stockings, or wearing shoes with a flimsy sole and foot problems have been linked to falls, instability, and fractures in older people [1–5]. Footwear is a modifiable risk factor for falls prevention [3] and appropriate shoes can enhance foot health by providing protection, support, and facilitating propulsion [4]. Despite the knowledge that people with stroke are at higher risk of falls and fractures than older people among the general population [6–10], few researchers have explored footwear among people with stroke [11,12]. Ng et al. in Australia [11] reported that among 30 people undergoing rehabilitation in the subacute stages post stroke (median 73 days post stroke onset), the majority wore slippers (53%), followed by close fitting outdoor shoes (30%) or walked barefoot (13%) indoors; whilst outdoors most wore close fitting shoes (*n* = 26, 87%). In a recent survey undertaken by our research team, 145 people with stroke responded. Of these, 35% wore slippers, 22% wore walking or oxford shoes, 11% walked in socks or barefoot, 5% reported wearing athletic shoes, and 2% wore surgical bespoke footwear indoors; outdoors 84% wore close fitting shoes [12].

Aspects of foot and ankle problems have previously been explored in people with stroke [13–17]. In comparison to a control group, people with stroke participants exhibited reduced sensation of the first metatarsophalangeal joint, greater foot pronation and reduced foot function; stroke fallers exhibited significantly greater foot pronation in comparison to non-fallers [15]. Most of the research published to date suggests that reduced foot range of motion and foot postures indicative of greater pronation are common post stroke [13–15]. In contrast, others reported foot postures indicative of supination particularly in the affected foot [16].

Despite growing awareness that foot and ankle problems can impact on balance performance and well-being in people with stroke [11,17], to date, no studies have been carried out exploring podiatrist assessed foot problems, footwear quality, and fit specific to people with chronic stroke. In this study, we built on our previous work [12,18] with the objective to explore indoor and outdoor footwear characteristics alongside podiatrist assessed foot problems in community dwelling people with stroke.

## Methods

### Governance

This project was part of a larger study exploring footwear and foot problems in relation to balance and falls in people with stroke and Parkinson disease (NIHR PB-PG-0212-27001). The larger study was split into four study components (a self-report survey [12], a podiatric foot (wear) assessment, an experimental movement analysis component exploring balance performance in indoor and outdoor shoes and a qualitative study component exploring the views and experiences of in relation to footwear choices [18]). This focus of this paper is reporting findings of the podiatric footwear assessment component. Ethical approval was granted (LREC: 14/SW/0078) and the study was sponsored by University Hospitals Southampton NHS Foundation Trust (R&D: RHM MED 1169). STROBE guidelines were considered during study conception, data analysis, and preparation of this manuscript [19].

### Study design and sample

In this exploratory cross-sectional observational study, participants were identified from hospital clinics and consultant lists, out-patient services and stroke support groups within the wider Southampton area for the survey component of our project. Full details of the recruitment to the survey and it its findings have been reported previously [12]. In the survey, respondents were invited to participate in the other study components and of the 145 survey respondents with stroke, 84 agreed to be contacted. All 84 people were screened during a phone call and invited to participate if they were able to mobilise at least 5 m (with or without a walking aid), were medically stable, able to answer simple questions, give informed consent and willing and able to attend a hospital appointment. Those with severe dysphasia, severe cognitive impairment who were unable to give informed consent, those with other neurological, unstable medical conditions and those who were bedbound were not invited to attend. Thirty people with stroke fulfilled the inclusion criteria and attended the podiatrist assessment.

### Procedure

A single assessment took place at the University of Southampton gait laboratory based within Southampton General Hospital. Participants were asked to bring their usual indoor (“the shoes they wear most often indoors”) and outdoor footwear (“the shoes they wear most often outdoors”) to the assessment, along with any ankle/foot orthosis routinely used. Written informed consent was obtained from all participants and descriptive data on age, past medical history, time since onset of stroke; side of weakness, fall history [8] health status post stroke [20], mobility [21], and balance confidence [22] were collected by an experienced physiotherapist. Health status post stroke was assessed using the Stroke Impact Scale (SIS), an interview administered questionnaire which involves questions about stroke related impairments and disabilities (including subsections on strength, memory, communication, activity, mobility, hand ability, participation, and overall recovery) from the patient’s point of view [20], scores range from 0 to 100 (lower scores indicating greater impairments). Mobility was assessed using the functional ambulation classification which categorises patients according to basic motor skills necessary for functional ambulation [21]. Balance confidence was assessed using the Tinetti Falls Efficacy Scale, a 10-item questionnaire used to explore people’s confidence in their ability to perform tasks without falling [22]. On this scale, total scores range from 10 to 100, with lower scores indicating confidence and higher scores indicating greater fear of falling and lower balance confidence. Retrospective fall history was assessed using the Fall Events Recall Aid [8] which involves a series of questions to identify whether participants had experienced any falls during the previous 12 months. For the study, a fall was defined as “an event that results in a person coming to rest unintentionally on the ground or other lower level, not as a result of a major intrinsic event or overwhelming hazard” [23].

### Footwear and foot status assessment

At the beginning of the assessment participants were asked if they felt they had any foot problems. Answers to this question were recorded as “self-reported foot problems”. Footwear and foot status was then assessed by a podiatrist to ascertain foot status, foot problems as well as the type, structural components, and fit of their footwear [24–28]. During the assessment, participants remained fully clothed (apart from removing their shoes and socks) and the assessment took place in sitting, standing and whilst lying comfortably on their back.

All shoes were assessed using the Footwear Assessment Tool [24] with particular emphasis on fixation, fit, heel counter stiffness, and support. During this assessment, shoes were classified as “indoor” or “outdoor” shoes and as “adequate” or “inadequate” depending on the footwear features. For example, “adequate” shoes had a small, a high collar, broad heel, thin and firm midsole, adequate means of fixation and adjustment and a textured slip-resistant outer sole. For example, if the fit of the shoe was tight on aspects of the foot, e.g., around bony prominences, squashing toes, or bunions (HAV) it was considered to narrow or too small. These features were recorded by the assessing podiatrist. In addition, a photograph was taken of all the shoes. The information about the footwear features and the photographs were then assessed by three podiatrists (the assessing podiatrist as well as two podiatrists who were not involved in the assessments and were blinded to participant and assessment outcomes). They made their decisions on the appropriateness of the footwear based on existing guidelines of adequate footwear features [28]. In cases of disagreement, a final decision was made by reaching consensus through discussion. The guidelines used to ascertain whether footwear was considered “adequate” was based on the expert group criteria for the recognition of healthy footwear [28]. Whilst expert opinion can only be considered low level evidence, in this study, the guidance was also combined with an expert podiatrist clinician performing the foot and footwear assessment.

We also included all measures that form part of the international consensus foot and ankle assessments (IMFAA) as recommended by Gates et al. [27]. The IMFAA includes observation of swollen/tender joints, assessing foot and ankle range of movement, muscle strength, and foot posture. Sensation tactile sensitivity of the plantar aspect of the first metatarsal phalangeal joint was assessed using a 10 g retractable monofilament (Bailey Instruments, Manchester, UK). The filament was applied to 10 sites until it bowed, and sensitivity was determined using a two alternative forced choice protocol according to current recommendations [24]. Foot status was assessed using the Manchester Foot Pain and Disability Index (MFPDI) [26]. To present findings in this study if a participant scored ≥1, they were considered to experience foot pain. For the podiatric assessment, we followed the tools definitions [24–28] and additional observations during the clinician assessment were noted.

### Statistical analysis

Analyses were conducted using Statistical Package for the Social Sciences version 28.0 software (SPSS Inc., Chicago, IL). Demographic and clinical characteristics of the sample, footwear and foot problems were described using summary statistics. The footwear specific characteristics and foot problems were presented as frequencies of occurrence. Pearson’s chi-squared analyses, Mann–Whitney’s *U* test and Spearman’s rank were used to explore associations between different footwear and foot status variables because data were not normally distributed. Statistical significance was judged at the two-sided 5% level.

## Results

### Participants

Thirty people with a mean time since stroke onset of 67 months attended the assessment (Table 1). The group presented with mild to moderate impairments (mean SIS mobility score = 68 ranging from 25 to 100 and overall recovery score = 64 ranging from 10 to 90). Twenty (67%) used a walking aid and six (20%) used an ankle foot orthosis to ambulate. Fifteen (50%) reported that they had or are currently receiving some footcare support following their stroke.

**Table 1.** Stroke participant characteristics (*n* = 30).

Variable		
Age (years)	Mean (SD)	68.2 (10.1)
	Min to max	46–84
Gender	Male/female	9/21
Side of weakness	Left	12 (40%)
	Right	15 (50%)
	Bilateral	2 (7%)
	None	1 (3%)
Walking aid use		20 (67%)
Ankle foot orthosis		6 (20%)
Months since stroke onset	Mean (SD)	66.8 (78.7)
	Min to max	5–384
Fall status	Non-faller	14 (47%)
	One-time-faller	7 (23%)
	Repeat-faller	9 (30%)
Indoor shoes age		0–6/6–12/>12 months
Fallers		2/3/11
No-fallers		5/3/14
Outdoor shoes age		0–6/6–12/>12 months
Fallers		4/1/11
Non-fallers		1/4/9
Living status	Alone/partner/family	10/17/3
SIS strength	Mean (SD)	57.1 (28.2)
0–100 higher scores better	Min to max	6–100
SIS memory	Mean (SD)	79.3 (22.4)
0–100 higher scores better	Min to max	18–100
SIS communication	Mean (SD)	80.2(22.1)
0–100 higher scores better	Min to max	18–100
SIS activity	Mean (SD)	64.8 (22.5)
0–100 higher scores better	Min to max	3–100
SIS mobility	Mean (SD)	68.1 (22.2)
0–100 higher scores better	Min to max	25–100
SIS hand ability	Mean (SD)	40.2 (40.8)
0–100 higher scores better	Min to max	0–100
SIS participation	Mean (SD)	65.7 (24.3)
0–100 higher scores better	Min to max	0–100
SIS overall recovery	Mean (SD)	64.4 (20.1)
0–100 higher scores better	Min to max	10–90
FPI		
Mean (SD)	Affected foot	4.2 (4.3) –6 to 11
Min to max	Unaffected foot	3.4 (3.0) –1 to 11
Tinetti Falls Efficacy Scale	Mean (SD)	27.6 (18.8)
0–100 higher scores worse	Min to max	10–91

SIS: Stroke Impact Scale; FPI: Foot Posture Index.

Figures are number (%) unless stated otherwise.

### Footwear style, fit, and structure

The footwear variety and podiatrist assessment of shoe fit, and features are shown in Table 2. Indoors, most participants wore slippers (*n* = 17, 57%, 95% CI 1.9–4.8), but 13 (43%) wore shoes indoors that were classified as outdoor shoes.

**Table 2.** Footwear characteristics.

ID	Indoor shoe style	Indoor shoe fit	Indoor shoe features^a^	Outdoor shoe style	Outdoor shoe fit	Outdoor shoe features^a^
1	Slipper	Good	Bad	Walking shoe	Good	Good
2	Slipper boot	Too short	Bad	Moccasin	Too short/shallow	Bad
3	Slipper	Too short	Bad	Walking shoe	Too long	Good
4/AFO	Slipper	Too short	Bad	Walking shoe	Too narrow	Good
5	Slipper	Too short	Good	Walking shoe	Too short	Bad
6	Walking shoe	Good	Good	Walking boots	Good	Good
7	Slipper	Too short	Good	Walking shoe	Good	Good
8	Backless slipper	Good	Bad	Walking shoe	Too short	Good
9	Backless slipper	Good	Bad	Walking shoe	Good	Good
10/AFO	Slipper	Good	Good	Waking shoe	Too short	Good
11	Slipper	Good	Good	Oxford shoe	Too long	Good
12	Slipper	Good	Bad	Sandals	Good	Good
13	Backless slipper	Too short	Bad	Oxford shoe	Good	Good
14/AFO	Surgical bespoke	Good	Good	Surgical bespoke	Too short	Good
15	Sandals	Good	Good	Walking shoe	Good	Good
16	Ballerina	Too short	Good	Sandal	Good	Good
17/AFO	Slipper boot	Good	Bad	Mary Jane	Good	Good
18	Slipper	Good	Bad	Mary Jane	Good	Good
19/AFO	Walking boot	Too long	Good	Walking boots	Too long/wide	Good
20	Sandals	Good	Good	Court shoe	Good	Good
21	Ballerina	Too short/narrow/shallow	Bad	Moccasin	Too short/shallow	Bad
22	Slipper	Too short/narrow shallow	Bad	Walking shoe	Good	Good
23	Athletic shoe	Too short/narrow/shallow	Good	Moccasin	Too shallow	Good
24	Court shoe	Too narrow/shallow	Bad	Walking shoe	Good	Good
25	Walking shoe	To narrow	Good	Oxford shoe	Good	Bad
26	Backless slipper	Too short/narrow/shallow	Bad	Moccasin	Too short/narrow/shallow	Bad
27	Walking shoe	Good	Bad	Walking shoe	Good	Bad
28	Moccasin	Good	Bad	Moccasin	Good	Good
29	Slipper	Good	Good	Walking shoe	Too wide	Good
30/AFO	Surgical bespoke	Too long	Good	Surgical bespoke	Too long	Good

AFO: ankle foot orthosis.

Figures are number (%) unless stated otherwise.

^a^ Good: broad heel, thin and firm midsole, adequate means of fixation and adjustment and a textured slip-resistant outer sole.

Fifty percent of the shoes worn indoors fitted poorly, and slipper type shoes tended to be too short or too narrow and shallow. Outdoors, walking shoes were the most common choice (*n* = 11, 46%) but again often fitted poorly (*n* = 14, 47%). Only 10 (33%) of the 30 participants wore well-fitting shoes both in- and outdoors whilst nine (30%) participants wore poorly fitting shoes both in- and outdoors. Among the six participants who regularly wore ankle foot orthoses of whom two wore bespoke shoes, indoor shoes fitted poorly for three participants, and outdoor shoes for five (Table 2).

In addition to fitting badly, indoor shoes were also more often classified as “inadequate” footwear choices (*n* = 16, 53%) by the three podiatrists. The characteristics that differed most between “inadequate” and “adequate” indoor footwear were lack of a means of fixation, no or minimal heel counter stiffness, and minimal midsole sagittal stability. A greater proportion of those who wore inadequate footwear indoors (12/16) had not received any footcare support following their stroke whilst 11/14 who had received some foot care support work adequate indoor shoes (*p* = 0.009). Forty percent of outdoor shoes (*n* = 24) were classified as “adequate”. It was observed that the six participants who wore inadequate outdoor shoes were all female.

### Foot problems, footwear, and falls

Whilst 19 (63%) participants self-reported foot problems, the podiatric assessment showed that all 30 of the participants had at least one foot problem (Table 3), with a mean number of 6.5 problems (95% confidence interval 5.4–7.7 problems). Although palpation abnormalities and impaired single heel raise are likely to impact on foot function and are useful clinical findings, one might argue that they may not be considered foot problems. When looking at the data that would indicate that one of the 30 participants who only presented with palpation abnormalities did not present with foot problems, but the remainder of the sample still present with two or more foot problems even after discounting these two factors. Those who self-reported foot problems also presented with a significantly greater number of foot problems during the podiatry clinician assessment component (*p* < 0.001, 95% CI 7.1–9.4) (see Table 3).

**Table 3.** Foot problems identified by the podiatrist using the IMFAA (*n* = 30).

Participant characteristics						IMFAA foot problem (see KEY)												
ID	Age (years)	Gender	Months since onset	Faller	Self report foot problems													
						Number	A	B	C	D	E	F	G	H	I	J	K	L
1	71	Male	17	No	No	1				x								
2	66	Female	8	No	No	3	x		x					x				
3	77	Male	91	Yes	No	3	x	x			x							
4	50	Female	16	Yes	No	4	x	x	x					x				
5	71	Female	14	No	No	4	x			x			x					x
6	66	Male	9	No	No	4	x				x		x			x		
7	76	Male	240	Yes	Yes	4	x		x				x	x				
8	59	Female	6	No	No	5	x	x	x	x					x			
9	73	Female	5	Yes	Yes	5	x			x		x			x		x	
10	57	Male	10	Yes	Yes	5	x	x	x							x	x	
11	67	Male	15	Yes	Yes	5		x		x		x	x		x			
12	60	Female	23	No	No	5	x	x	x	x					x			
13	78	Male	88	Yes	No	5	x	x	x	x						x		
14	71	Female	77	No	No	5	x	x	x						x		x	
15	59	Female	77	Yes	No	6	x	x	x		x	x			x			
16	74	Female	44	No	No	7	x	x	x		x	x	x				x	
17	77	Female	51	Yes	Yes	7	x	x	x	x	x			x	x			
18	52	Female	132	No	Yes	7	x	x	x	x	x	x	x					
19	66	Male	115	Yes	Yes	7	x	x	x	x		x	x	x				
20	77	Female	94	Yes	Yes	7	x	x	x	x	x		x	x				
21	77	Female	30	No	Yes	7	x	x		x		x	x	x	x			
22	46	Female	10	No	Yes	7	x		x	x	x	x	x	x				
23	84	Female	39	Yes	Yes	8	x	x	x	x	x		x		x	x		
24	81	Female	96	Yes	Yes	8	x	x	x		x	x		x		x		x
25	78	Female	64	Yes	Yes	8	x	x	x	x	x	x			x	x		
26	64	Female	24	No	Yes	8	x	x	x	x		x		x	x	x		
27	57	Female	72	Yes	Yes	8	x	x	x	x	x	x	x			x		
28	84	Female	101	No	Yes	9	x	x	x	x	x	x	x	x			x	
29	67	Male	51	No	Yes	9	x	x	x	x	x	x			x	x		x
30	61	Female	384	Yes	Yes	12	x	x	x	x	x	x	x	x	x	x	x	x

KEY (IMFAA: International Musculoskeletal Foot and Ankle Assessment).		
	Foot problem	Podiatrist foot problems (*n* = 30), number (%)
A	Impaired single limb heel raise	28 (93%)
B	Impaired range of foot and ankle movement	23 (77%)
C	Gait abnormalities	23 (77%)
D	Palpation abnormalities	19 (63%)
E	Hallux valgus	15 (50%)
F	Pain	15 (50%)
G	Impaired sensation	14 (47%)
H	Reduced muscle strength	13 (43%)
I	Swollen joints	12 (30%)
J	Skin and nail changes	10 (33%)
K	Toe deformities	7 (23%)
L	Foot morphologies	4 (13%)

The “x” links specific problems for each participant to a key outlined below the table so as to enable readers to identify which foot problems and combination of foot problems were experienced.

**Figure 1.**
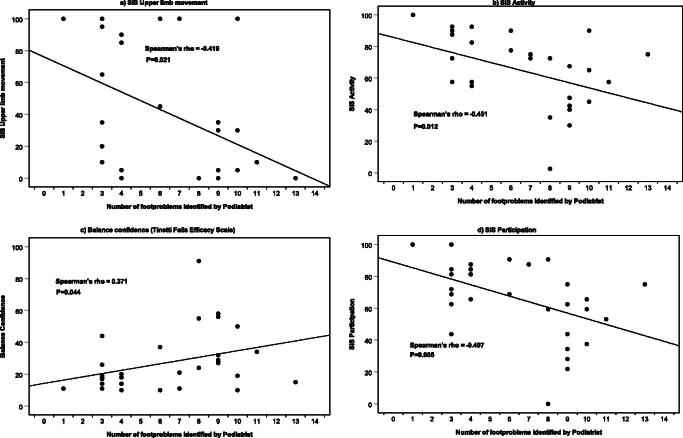
Four scatter plots showing the correlation between number of foot problems identified by the podiatrist during the assessment and three different Stroke Impact Scale sections and balance confidence as measured using the Tinetti Falls Efficacy Scale.

The number of foot problems was associated with poorer balance confidence, activity levels, participation, and upper limb movement ability (see Figure 1) indicating that we observed that those who presented with greater levels of disability, poorer balance, and reduced activity levels were presented with a greater number of foot problems during the clinician podiatric foot assessment.

Findings from the fall-events recall aid showed that over half of the participants reported falls (*n* = 16, 53%) and nine (30%) had fallen repeatedly. Participants who reported falls experienced a greater levels of stroke related disability (*p* = 0.006) as they achieved lower scores on the SIS (55.1 (SD 22.8); 95% CI 42.9–67.2) than non-fallers (75.1 (SD 8.6); 95% CI 70.1–80.1). A significantly greater proportion of fallers (13/16) reported foot problems (*p* = 0.029) in comparison to non-fallers (4/14). Of those who experienced falls, nine wore adequate indoor shoes and seven inadequate indoor shoes; however, nine of the non-fallers also wore inadequate indoor shoes. Fourteen fallers and 10 non fallers wore adequate outdoor shoes. Among nine fallers and six non-fallers indoor shoes did not fit well and among seven fallers and seven non-fallers outdoor shoes fitted poorly. Eleven fallers and six non-fallers wore indoor shoes that they had worn for longer than 12 months and nine non-fallers and 11 fallers had been wearing their outdoor shoes for longer than 12 months.

## Discussion

To our knowledge, this is the first study to explore what community dwelling people with chronic stroke usually wear on their feet indoors and outdoors rather than relying on self-report. In this study, we built upon the self-reported foot problems from our previously published survey [12] with a clinician assessed component that included a standardised podiatric assessment of footwear type, fit, quality, and foot problems [27] because self-reported foot problems can differ from clinician assessed reports.

Our findings are in line with our previously published survey and qualitative study findings, suggesting that footwear choices and foot problems challenge people with stroke [12,18]. In the present study, many community-dwelling people with stroke wore poorly fitting and inappropriate shoes, all had foot problems, and a high percentage reported instability and falls.

Many participants wore slippers indoors (*n* = 17, 57%), slightly higher than self-reported in our larger survey (35%) [12], but in agreement with previous studies in stroke and the general elderly [6,28]. Together the evidence suggests that most people continue to wear slipper type shoes or generally unsupportive footwear indoors [12,29–31] despite the increased risk of foot problems and falls. Findings from our related previously published qualitative study component showed that men typically always wore slippers indoors because they provide comfort and warmth, are easy to get on and off, and accommodate swollen feet; whereas women were concerned about the shortcomings of slippers and wore them for shorter periods during the day [18]. Others reported that older people often wear slippers around the home all day and replace them infrequently [32].

Fewer outdoor shoes were classified as “inadequate” but all those that were, were worn by women. These findings are supported by a recent cross-sectional study among an older inpatient population [33] where female gender was identified as one of the most consistent factors associated with wearing outdoor shoes that are not recommended for supporting balance and mobility.

Although 70% of respondents in our previous survey [12] indicated that they felt their current indoor shoes “were right for them”, when assessed by a podiatrist we found that only 50% of the shoes worn by participants in the current study fitted well. A systematic review concluded that between 63 and 73% people wore poorly fitting shoes, highlighting that this is a very common problem [34]. Worryingly even bespoke shoes supplied and worn with a foot orthosis did not fit well in all cases. Evidence shows that shoe fit is important [34] and that correctly fitted ankle-foot orthosis can have a beneficial effect on gait post stroke [35]. It is argued that poorly fitted shoes might not allow the ankle foot orthosis to fulfil its intended purpose. Poorly fitting shoes have been linked to the development of foot problems including toe deformities, pain, skin lesions, hallux valgus, corns, and calluses [34].

The objective podiatric foot status assessment in our study revealed that all participants presented with foot problems (median 7, range 1–13); 50% reported pain. The common foot problems identified in our sample using the IMFAA [27] were difficulty or inability to perform a single limb heel raise (93%), impaired range of foot and ankle movement (77%), palpation abnormalities (63%), and hallux valgus (50%). One might argue that palpation abnormalities and single limb heels raise problems are clinical symptoms rather than pure foot problems. However, even after removing one participant who only presented with palpation abnormalities and after discounting all reported single limb raise difficulties, 29 participants still presented with two or more other foot problems.

The average Foot Posture Index scores for stroke participants in this study was 3 (–2 to 11), reflecting findings of some other studies [13–16] and the general population [36]. The percentage of individuals in the general population aged 65 years and older who suffer from foot problems has been found to vary between 30% and 86% [2,37–46]. The variation, in part, may be due to the different case definitions across the constituent studies, and that most stroke specific studies have included only some aspects, and none used exactly the same assessments. Our data suggest that the prevalence of some foot problems among people with stroke appears to be higher in comparison to the general elderly population with 50% presenting with hallux valgus or pain and 47% presenting with impaired sensation. For example, prevalence of hallux valgus among an elderly population has been estimated at 36% [41–44], foot pain was found in 24–30% of older adults [2,43], swelling in 26% and non-diabetic loss of sensation was reported in 5% [40].

In the present study, we observed associations indicating that those with greater levels of disability, poorer balance, and reduced activity levels more often presented with a greater number of foot problems during the clinician podiatric foot assessment. Whilst it is not possible to make any causal claims, these observations agree with similar observations made in other studies that explored some aspects of foot problems in people with stroke [13–17]. Among older people foot problems such as impaired sensation and reduced foot and ankle strength are significant independent predictors of functional ability and balance [45] and reduced range of foot and ankle movement and strength, pain and hallux valgus have been associated to poor balance and falls in older adults [2,45–48]. Poor upper limb function was also associated with greater number of foot problems in this study. Poor upper limb function in people with stroke has been previously identified as a predictor of falls [49]. Clinical experience also suggest that limited arm movement might reduce ability for foot self-care and reliance on footwear that can be put on and taken off easily. Those with foot problems were less active and had reduced community participation. This corresponds with similar findings among older people, where authors also reported a negative impact on quality of life [50].

## Limitations

Whilst attempting to reach a representative sample of community dwelling people with stroke in the previously published larger (survey) component of the study [12], it is acknowledged that selection bias was likely introduced as participants for the present study were drawn from survey respondents who agreed to be contacted. It is likely that the more able and highly motivated people with stroke agreed to take part as participation required attending an in-person hospital-based assessment. Thus, it is highly unlikely that the resulting sample can be considered representative. Whilst potential associations were explored and reported in this study no causal claims were made as observations are reported based on a one-off measurement. These limitations combined with the other inherent weaknesses of cross-sectional observational designs further limit generalisability. However, considering the likelihood that more able stroke participants agreed to participate, the high percentage of participants who experienced foot problems that were identified during the clinician podiatric assessment and the fact that so many wore unsupportive poorly fitting shoes, could suggest that the actual percentage of people with stroke experiencing foot problems who wear inappropriate footwear is even higher. On the other hand, it is also possible that more people with stroke who experienced foot problems or had footwear concerns agreed to take part in the study hoping for advice and support, so findings must be interpreted with caution. Whilst the best currently available evidence podiatric assessment tools were employed in this study, it is acknowledged that these tools are based on low level evidence [24–28] and that there is still a lack of standard validated assessment tools for podiatric foot assessments. To counteract these limitations, we included a clinician podiatric assessment as an expert podiatrist has the best level of experience in assessing foot problems and fit of footwear. Therefore, this paper may be considered as a descriptive account based on findings observed in this sample of people with stroke that forms a baseline for future work. It is not yet known whether greater stroke severity and greater levels of disability increase the risk of developing foot problems post stroke or whether a greater number of foot problems develop post stroke that negatively impact balance and mobility post stroke further increasing disability; this warrants further exploration in a longitudinal or controlled intervention study.

## Clinical implications

Emerging evidence suggests that people with stroke currently feel unsupported and perceive a lack of professional advice of footwear choices [12,18]. These finding are also reflected in the steady decline in the number of podiatrists [47,48]. Ideally all people with stroke would have access to NHS podiatric support. However, considering acknowledged shortages in podiatrist provision this would not be realistic or achievable. Our recommendation is to improve access to information about good shoe characteristics for people with stroke, ideally when they have contact with other clinicians [28]. However, access to this information on its own this is unlikely to resolve the problem. Shoe purchasing decisions are often based on comfort and convenience and low cost; most older people and people with stroke were unwilling or unable to buy more expensive shoes [18,30–32]. Better and more tailored support is needed for people with stroke, including better condition specific information on appropriate footwear [31] and encouragement and support that new shoe purchases should include measuring feet to ensure a good fit [34]. Dedicated novel interventions targeted at including patient centred footwear discussions [51–53] as well as upskilling other clinicians or third sector resources are needed to give people with stroke better options and promote new habit formation [54]. Without a new and better targeted approach many patients will continue to wear slippers and other unsupportive badly fitting shoes, despite the risks and negative health consequences.

## Conclusions

Many people with stroke wear slippers and unsupportive poorly fitting shoes; a high proportion present with foot problems that negatively impacted on their balance confidence, activity, and community participation. Further investigation is warranted to explore the most effective pathways to provide appropriate support in relation to foot care and for helping people with stroke to choose and wear appropriate supportive and well-fitting indoor and outdoor shoes.
